# Association of tumor necrosis factor-alpha promoter variants with risk of HPV-associated oral squamous cell carcinoma

**DOI:** 10.1186/1476-4598-12-80

**Published:** 2013-07-19

**Authors:** Lei Jin, Erich M Sturgis, Yang Zhang, Zhigang Huang, Xicheng Song, Chao Li, Qingyi Wei, Guojun Li

**Affiliations:** 1 Department of Stomatology, Jinling Hospital, School of Medicine, Nanjing University, Southern Medical University, Nanjing 210002, China; 2 Department of Head and Neck Surgery, The University of Texas MD Anderson Cancer Center, Houston, TX 77030, USA; 3 Department of Epidemiology, The University of Texas MD Anderson Cancer Center, Houston, TX 77030, USA; 4 Department of Otolaryngology Head and Neck Surgery, Beijing Tongren Hospital, Capital Medical University, Beijing 100730, China; 5 Department of Otorhinolaryngology and Head and Neck Surgery, Yuhuangding Hospital of Qingdao University, Yantai 264000, China; 6 Department of Head & Neck Surgery, Sichuan Cancer Hospital & institute, Chengdu, Sichuan 610041, China

**Keywords:** *TNF*-*α*, Polymorphism, HPV infection, Oropharyngeal cancer, Case–control study

## Abstract

**Background:**

Tumor necrosis factor alpha (TNF-α) plays an important role in inflammation, immunity, and defense against infection and clearance of human papillomavirus (HPV). Thus, genetic variants may modulate individual susceptibility to HPV-associated oral squamous cell carcinoma (OSCC).

**Methods:**

In this study we genotyped four common single nucleotide polymorphisms (SNPs) in the *TNF*-*α* promoter [ −308G > A(rs1800629), -857C > T (rs1799724), -863C > A (rs1800630), and -1031T > C (rs1799964)] and determined HPV16 serology in 325 OSCC cases and 335 matched controls and tumor HPV status in 176 squamous cell carcinomas of the oropharynx (SCCOP) patients. Univariate and multivariable logistic regression models were used to calculate odds ratios (ORs) and 95% confidence intervals (CIs).

**Results:**

We found that HPV16 seropositivity alone was associated with an increased risk of OSCC (OR, 3.1; 95% CI, 2.1–4.6), and such risk of HPV16-associated OSCC was modified by each SNP. Patients with both HPV16 seropositivity and variant genotypes for each SNP had the highest risk when using patients with HPV16 seronegativity and a wild-type genotype as a comparison group. Moreover, similar results were observed for the combined risk genotypes of four variants and all such significant associations were more pronounced in several subgroups, particularly in SCCOP patients and never smokers. Notably, the combined risk genotypes of four variants were also significantly associated with tumor HPV-positive SCCOP.

**Conclusion:**

Taken together, these results suggest that *TNF*-*α* SNPs may individually or, more likely, jointly affect individual susceptibility to HPV16-associated OSCC, particularly SCCOP and never smokers. Validation of our findings is warranted.

## Introduction

Oral squamous cell carcinoma (OSCC) includes cancers arising from the oropharynx and oral cavity. In the United States, an estimated 40 250 new cases of OSCC and 7850 deaths from OSCC are expected in 2012 [1]. Tobacco and alcohol are well-established risk factors for OSCC. Corresponding with the decrease in tobacco use in the United States, the incidence rate of OSCC has declined in the past two decades, while the incidence of a subgroup of OSCC, squamous cell carcinomas of the oropharynx (SCCOP), has increased in recent years, particularly in young adults and nonsmokers and nondrinkers. The rising incidence of SCCOP in the United States is likely a consequence of persistent infection with human papillomavirus (HPV), predominantly high-risk HPV type 16 (HPV16). The overall rise in SCCOP incidence during 1984 to 2004 is largely explained by the increasing incidence of HPV-positive cancers, whereas incidence of HPV-negative cancers declined. Consequently, HPV prevalence in oropharyngeal tumors increased substantially from 16.3% during the 1980s to 72.7% during the 2000s [2-4]. Population-level incidence of HPV-positive SCCOP increased by 225% from 1988 to 2004 (from 0.8 per 100,000 to 2.6 per 100,000), while the incidence for HPV-negative SCCOP decreased by 50% (from 2.0 per 100,000 to 1.0 per 100,000) [2]. In addition to HPV infection, it is likely that other, as-yet-unknown genetic factors in inflammation and immune response pathways may also be associated with the risk of HPV-associated OSCC, particularly SCCOP.

The host immune responses and chronic inflammation have been shown to be biologically important risk factors for HPV-related carcinogenesis, and increased duration of persistent HPV infection may be influential in determining disease development, indicating the importance of the host immune response to HPV clearance and HPV-related carcinogenesis. Cytokines are a group of host immune products involved in inflammation, immunity, defense against HPV infection, and modulation of HPV clearance. Tumor necrosis factor-alpha (TNF-α) is a multifunctional cytokine in the host response to inflammation, immunity, and the defense against viral infections [5]. TNF-α may be involved in carcinogenesis through induction of proliferation, invasion, and metastasis because it may have both tumor-necrotic and tumor-promoting activities [6]. Increased expression of TNF-α has been observed in association with HPV infection in both normal cervical tissues and cervical cancers [7], supporting the importance of TNF-α in response to HPV and to subsequent carcinogenesis. TNF-α also has been found to repress expression of oncogenes *E6* and *E7* at the translation level in HPV16-immortalized human cells [8], induces apoptosis and growth arrest in normal and HPV16-infected cells [9,10], and stimulates inflammatory response through upregulation of chemokines and other inflammatory regulators [8,11]. On the other hand, HPV16 could modulate the effect of TNF-α because E6 and E7 expressions are associated with resistance to the TNF-α-mediated apoptosis and TNF-α-induced anti-proliferative effect, suggesting an important role for TNF-α in HPV-associated carcinogenesis [9,12]. In contrast, TNF-α also promotes tumor activity by stimulating the proliferation of cervical cells immortalized and transformed by HPV [13,14]. Therefore, TNF-α expression levels may influence HPV infection and subsequent HPV-associated cancer development.

Given the potential roles of TNF-α as an important and pleiotropic cytokine that plays a critical role in immune regulation through prominent anti-inflammatory and immunoregulatory activities (11), its genetic variants may affect the host immune system and HPV infection and, subsequently, the HPV-associated cancer development. Several single nucleotide polymorphisms (SNPs) in the promoter region of the *TNF*-*α* gene may have putatively functional changes and thus may affect an individual’s susceptibility to cancer. For example, the SNP at site −308 (G➔A, rs1800629) was shown to affect the transcriptional activity of *TNF*-*α* because that SNP’s A allele was associated with increased *TNF*-*α* production *in vitr*o [15]. In addition, an enhanced transcriptional activity was associated with the minor alleles of SNPs at −857 (C➔T, rs1799724), -863 (C➔A, rs1800630), and −1031 (T➔C, rs1799964) in response to stimuli [16]. Therefore, we hypothesized that these promoter SNPs may be associated with risk of HPV-associated OSCC, particularly SCCOP.

Several studies have evaluated associations of the above-mentioned *TNF*-*α* promoter SNPs with the risk of several types of cancer, including those of the cervix, stomach, and colon and rectum and non-Hodgkin lymphoma [17-20]. Studies on associations between *TNF*-*α* variants at −308 and −1031 and risk of oral cancer also been reported, but the results between those studies were not consistent and those studies also had relatively small sample sizes [20-26]. More importantly, studies specifically evaluating the effect of *TNF*-*α* promoter SNPs on the risk of HPV-associated OSCC are lacking. Therefore, in the present study, we genotyped *TNF*-*α* promoter SNPs [−308G > A (rs1800629), -857C > T (rs1799724), -863C > A (rs1800630), and -1031T > C (rs1799964)] and evaluated their associations with risk of HPV16-associated OSCC in 325 OSCC patients and 335 cancer-free controls, all of whom were non-Hispanic whites.

## Materials and methods

### Study populations

In this case–control study, the 325 cases were patients with newly diagnosed, histopathologically confirmed and untreated OSCC. The details of recruitment and the inclusion criteria for these cases were described previously [27]. Briefly, these cases had been consecutively recruited during the period between May 1996 and May 2002 at The University of Texas MD Anderson Cancer Center as part of an ongoing molecular epidemiologic study of head and neck cancers. During that same period, the controls had been selected from a pool of cancer-free subjects recruited from the Kelsey-Seybold Foundation, a multispecialty physician practice with multiple clinics throughout the Houston metropolitan area, as well as from healthy visitors who had accompanied cancer patients to outpatient clinics at MD Anderson but who were genetically unrelated to the patients. The 335 controls were frequency-matched to the patients by age (±5 years), sex, and smoking and drinking status. Only non-Hispanic whites were included as controls because most of the cancer patients recruited were non-Hispanic whites. Approximately 95% of eligible incident cases and 78% of eligible controls had agreed to participate in the study. The study received approval from the institutional review boards of both MD Anderson and Kelsey-Seybold, and all study subjects signed an informed consent when approached for recruitment. Subjects who had smoked more than 100 cigarettes in their lifetimes were categorized as “ever-smokers,” and others as “never-smokers.” Subjects who had consumed alcoholic beverages at least once a week for more than 1 year previously were categorized as “ever-drinkers,” and others as “never-drinkers”.

### HPV16 serological testing

For the current study, serum samples from each subject were tested for anti-HPV16 (antibody against HPV16) by a standard enzyme-linked immunosorbent assay with HPV16 L1 virus-like particles generated from recombinant baculovirus-infected insect cells, as described previously [28]. Ten percent of the samples were randomly chosen for re-testing, and the results were in 100% concordance with those of the initial assays.

### Tumor HPV16 determination

Paraffin-embedded tissues were tested for HPV16 DNA using polymerase chain reaction (PCR)-based, type-specific assays with modification and quality control for the E6 and E7 regions [3,4]. Assays of the samples were run in triplicate, with positive and negative controls (Siha and TPC-1 cell lines, respectively). β-actin was used as a DNA quality control. Specificity for HPV16 E6 and E7 was confirmed by Southern blot analysis of paraffin-embedded tissue samples using a Roche Diagnostics labeling and hybridization system (Roche Applied Science, Indianapolis, IN) [4]. HPV16 E6 and E7 specificity was confirmed by retesting 10% of the samples using restriction digestion of the PCR products with Ban*II* and Msp*I* to verify the presence of E6- and E7-specific fragments. The results of both methods were 100% concordant.

### TNF-α genotyping

For this study, we extracted genomic DNA from a leukocyte cell pellet using the QIAamp DNA Blood Mini Kit (QIAGEN Inc., Valencia, CA) in accordance with the manufacturer’s instructions. The polymerase chain reaction-restriction fragment length polymorphisms (PCR-RFLP) method was used for genotyping the selected SNPs. Information on primers and endonucleases used are provided in Table 1. Genotyping was performed by laboratory personnel blinded to the case–control status. Repeated analysis was performed on a randomly selected subset of 10% of the samples, and the results were in 100% concordance with the initial analysis.

**Table 1:** **Summary of *TNF***-***α *PCR**-**RFLP analysis**

**Variants**	**Primer sequence (5**′ **to 3′)**	**PCR products**	**Anneal T**		**Allele**	
	**Upper-forward; lower-reverse**		**(°C)**	**Enzymes used**	**determination**	
*NF*-*α*-308G > A (rs1800629)	AGGCAATAGCTTTTGAGGGCCATG	80 bp	60	Sty I	Intact	:-308GG
	TCTTCTGGGCCACTGACTGAT					
					58 bp + 22 bp	:-308GA + AA
*TNF*-*α*-857C > T (rs1799724)	CGAGTATGGGGACCCTCCCATTAA	100 bp	57	Ase1	Intact	:-857CC
	ACGTCCCCTGTATTCCATACC					
					78 bp + 22 bp	:-857CT + TT
*TNF*-*α*-863C > A (rs1800630)	GACCACAGCAATGGGTAGGAGA	86 bp	60	Bsl I	Intact	:-863CC
	TCTACATGGCCCTGTCCTCGT					
					64 bp + 22 bp	:-863CA + AA
*TNF*-*α*-1031T > C (rs1799964)	AGAGCTGTGGGGAGAACAAA	247 bp	62	Bbs I	Intact	:-1031TT
	CTCCTACCCATTGCTGTGGT					
					127 bp + 120 bp	:-1031TC + CC

### Statistical analysis

Statistical analyses were performed using the SAS software, version 9.2 (SAS Institute Inc., Cary, NC). All tests were two-sided, and a *P* value of < 0.05 was considered the cutoff for statistical significance. We used χ^2^ tests to examine differences between the patients and controls in the distributions of demographic variables, smoking status, drinking status, HPV16 status, and genotypes. We evaluated both the association of HPV16 status and TNF-α genotypes, individually and in combination, with the risk of OSCC by computing odds ratios (OR) and their 95% confidence intervals (CI), using both univariate and multivariable logistic regression analyses. The analyses of joint effects were further stratified by tumor site and patient age and smoking and drinking status.

## Results

### Demographics and risk factors for the study population

The demographics and OSCC risk factors for the 325 patients and 335 controls are shown in Table 2. Among the 325 patients, 188 (57.8%) had SCCOP, and 137 (42.2%) had oral cavity cancers. Age, sex, and smoking and drinking status did not differ significantly between patients and controls as a result of frequency matching. However, HPV16 seropositivity was more common in patients than in controls (*P* < 0.001) and was associated with a 3.1-times higher risk of OSCC than in controls (OR, 3.1; 95% CI, 2.1–4.6).

**Table 2:** Demographic and risk factors in patients and controls and their association with risk of OSCC

**Variables**	**Patients *(n = 325)***		Controls (n = 335)^*^	
	**No.**	**%**	**No.**	**%**
Age (years)				
≤ 40	31	9.5	27	8.1
41 – 55	126	38.8	105	31.3
56 – 70	119	36.7	154	46.0
> 70	49	15.0	49	14.6
Sex				
Male	241	74.2	269	80.3
Female	84	25.8	66	19.7
Tobacco smoking				
Ever	227	69.8	239	71.3
Never	98	30.2	96	28.7
Alcohol drinking				
Ever	250	76.9	240	71.6
Never	75	23.1	95	28.4
HPV16 serology^a^				
Negative	225	69.2	293	87.5
Positive	100	30.8	42	12.5

^*^The cases and controls were adequately frequency-matched.

^a^significantly different in HPV16 serological status between cases and controls.

### Joint effect of HPV16 seropositivity and TNF-α SNPs on the risk of OSCC

Table 3 shows the association between *TNF*-*α* genetic variants and the risk of HPV16-associated OSCC. Corresponding to each SNP, HPV16-seropositive individuals carrying variant genotypes of *TNF*-*α*-308A, *TNF*-*α*-857T, *TNF*-*α*-863A, or *TNF*-*α*-1031C had a higher risk of OSCC after adjustment for age, sex, and smoking and drinking status than did individuals with both HPV16-seronegativity and the homozygous wild-type genotype. For each SNP, using individuals with both HPV16 seronegativity and the homozygous wild-type genotype as the reference group, the risk of OSCC increased among individuals with both variant genotypes and HPV16 seronegativity, both wild-type genotype and HPV16 seropositivity, and both variant genotypes and HPV16 seropositivity, respectively. For example, compared with individuals with the *TNF*-*α*-308 GG genotype and HPV16 seronegativity, the risk increased among those with GA or AA genotypes and HPV16 seronegativity (OR, 1.3; 95% CI, 1.0–1.9), GG genotype and HPV16 seropositivity (OR, 2.5; 95% CI, 1.3–4.8), and GA or AA genotypes and HPV16 seropositivity (OR, 4.8; 95% CI, 2.7–8.4). The similar results were found for other SNPs (Table 3). Furthermore, all such significant associations were particularly evident for SCCOP as opposed to oral cavity cancers.

**Table 3:** **Associations of *TNF***-**α SNPs with risk of HPV16**-**associated OSCC**

		**Patients**		**Controls**		**Adjusted OR (95% CI)**^**1**^		
		***(n = 325)***		***(n = 335)***				
**Variables**	**HPV16 Status**	**No.**	**%**	**No.**	**%**	**OSCC**	**SCCOP**	**Oral cavity**
*TNF*-*α*-308								
GG	−	70	21.5	109	32.5	1.0	1.0	1.0
GA + AA	−	155	47.7	184	54.9	1.3 (1.0 − 1.9)	1.4 (1.0 − 2.3)	0.4 (0.1 − 1.5)
GG	+	31	9.5	19	5.7	2.5 (1.3 − 4.8)	4.6 (2.2 − 9.5)	1.1 (0.7 − 1.8)
GA + AA	+	69	21.2	23	6.9	4.8 (2.7 − 8.4)	9.3 (4.9 − 17.6)	1.2 (0.5 − 2.8)
*TNF*-*α*-857								
CC	−	198	60.9	261	77.9	1.0	1.0	1.0
CT + TT	−	27	8.3	32	9.6	1.2 (0.7 − 2.1)	1.4 (0.7 − 2.7)	0.7 (0.3 − 1.4)
CC	+	85	26.2	38	11.3	3.0 (2.0 − 4.7)	5.6 (3.5 − 9.0)	1.0 (0.5 − 1.9)
CT + TT	+	15	4.6	4	1.2	5.3 (1.7 − 16.2)	9.3 (3.0 − 29.6)	1.5 (0.3 − 8.6)
*TNF*-*α*-*863*								
CC	−	66	20.31	115	34.3	1.0	1.0	1.0
CA + AA	−	159	48.9	178	53.1	1.6 (1.1 − 2.3)	1.8 (0.8 − 2.2)	0.8 (0.3 − 2.4)
CC	+	34	10.5	19	5.7	3.1 (1.6 − 5.9)	5.2 (2.5 − 10.5)	1.7 (1.0 − 2.7)
CA + AA	+	66	20.3	23	6.9	5.3 (3.0 − 9.4)	8.5 (4.6 − 16.0)	1.3 (0.5 − 3.2)
*TNF*-*α*-1031								
TT	−	71	21.9	115	34.3	1.0	1.0	1.0
TC + CC	−	154	47.4	178	53.1	1.4 (1.0 − 2.0)	1.6 (1.0 − 2.6)	0.7 (0.2 − 2.3)
TT	+	31	9.5	17	5.1	3.1 (1.6 − 6.1)	6.0 (2.8 − 12.4)	1.2 (0.8 − 2.0)
TC + CC	+	69	21.2	25	7.5	4.5 (2.6 − 7.9)	9.0 (4.8 − 16.7)	1.0 (0.4 − 2.3)

^1^Adjusted for age, sex, and smoking and alcohol drinking status.

To assess the combined effect of *TNF*-*α* SNPs on the risk of HPV16-associated OSCC, individuals were further categorized into three groups according to their number of risk genotypes on the basis of results of OSCC risk associated with each individual SNP: 1) a low-risk group (individuals carrying 0–1 risk genotypes); 2) a medium-risk group (individuals carrying 2 risk genotypes); and 3) a high-risk group (individuals carrying 3 or 4 risk genotypes) as shown in Table 4. When we used HPV16-seronegative individuals from the low-risk group as the reference group, we found that the risk of OSCC significantly increased among HPV16-seronegative individuals categorized as high-risk (OR, 1.8; 95% CI, 1.1–2.8), among HPV16-seropositive individuals categorized as low-risk (OR, 1.5; 95% CI, 1.0–2.9), among HPV16-seropositive individuals categorized as medium-risk (OR, 2.7; 95% CI, 1.3–5.8), and among HPV16-seropositive individuals categorized as high-risk (OR, 8.5; 95% CI, 3.7–19.4). The exception was HPV16-seronegative individuals from the medium-risk group (OR, 1.0; 95% CI, 0.3–1.0). Again, all aforementioned associations were particularly pronounced for SCCOP as opposed to oral cavity cancers.

**Table 4:** **Joint effect of HPV16 serology and the combined risk genotypes of *TNF***-***α *SNPs on risk of OSCC**

**Risk groups**^**1**^	**HPV16 status**	**Patients**		**Controls**		**Adjusted OR (95% CI)**^**2**^		
		***(n = 325)***		***(n = 335)***				
		**No.**	**%**	**No.**	**%**	**OSCC**	**SCCOP**	**Oral cavity**
Low-risk group	−	61	18.8	81	24.2	1.0	1.0	1.0
Medium-risk group	−	53	16.3	129	38.5	1.0 (0.3 − 1.0)	1.0 (0.3 − 1.1)	0.5 (0.3 − 1.0)
High-risk group	−	111	34.2	83	24.8	1.8 (1.1 − 2.8)	1.8 (1.0 − 3.3)	1.6 (0.9 − 2.8)
Low-risk group	+	27	8.3	13	3.9	1.5 (1.0 − 2.9)	2.9 (1.4 − 6.2)	0.7 (0.2 − 2.4)
Medium-risk group	+	23	7.1	21	6.3	2.7 (1.3 − 5.8)	4.9 (2.2 − 11.2)	0.2 (0.0 − 1.0)
High-risk group	+	50	15.4	8	2.4	8.5 (3.7 − 19.4)	15.8 (6.5 − 38.0)	2.3 (0.7 − 6.9)

^1^Low-risk group: individuals carrying 0–1 risk genotypes; medium-risk group: individuals carrying 2 risk genotypes; and high-risk group: individuals carrying 3 or 4 risk genotypes.

^2^Adjusted for age, sex, and smoking and alcohol drinking status.

### Stratification analysis of the joint effect of HPV16 serology and combined risk genotypes of TNF-α on risk of OSCC

We further evaluated the association between the combined risk genotypes of *TNF*-*α* and risk of HPV16-associated OSCC stratified by age and smoking status. As shown in Table 5, the joint effect of both HPV16 serology and combined risk genotypes of *TNF*-α on risk of OSCC was greater in young (less than 50 years old) subjects than in subjects older than 50 years or older. Specifically, a 22.6-times higher risk of OSCC was found in HPV16-seropositive young subjects in the high-risk genotype group, compared with a 5.7-times higher risk of OSCC in HPV16-seropositive older subjects in the high-risk group. Similarly, as shown in Table 6, never-smokers were at greater risk of OSCC than were ever-smokers, respectively. Specifically, HPV16-seropositive individuals in the high-risk genotype group had an OR of 35.6 in never-smokers versus an OR of 4.8 in ever-smokers. Moreover, such risk estimates stratified by age and smoking status were even more pronounced for SCCOP (Tables 5 and 6), whereas we did not observe similar associations among patients with oral cavity cancers (data not shown).

**Table 5:** **Joint effect of HPV16 serology and the combined risk genotypes of *TNF***-***α *SNPs on risk of OSCC**, **stratified by age**

**Risk groups**^**1**^	**HPV16 status**	**Patients**		**Controls**		**Adjusted OR (95% CI)**^**2**^	
		***(n = 325)***		***(n = 335)***			
		**No.**	**%**	**No.**	**%**	**OSCC**	**SCCOP**
Young (aged < 50 years)		(*n* = 87)		(*n* = 87)			
Low-risk group	−	12	13.8	29	33.3	1.0	1.0
Medium-risk group	−	19	21.8	35	40.2	1.2 (0.5 − 3.1)	1.1 (0.3 − 4.5)
High-risk group	−	24	27.6	13	14.9	4.1 (1.5 − 11.5)	5.5 (1.4 − 21.2)
Low-risk group	+	7	8.1	5	5.8	4.7 (1.1 − 19.7)	10.1 (2.0 − 52.3)
Medium-risk group	+	9	10.3	3	3.5	8.9 (1.8 − 43.5)	16.6 (2.9 − 6.1)
High-risk group	+	16	18.4	2	2.3	22.6 (4.2 − 121.3)	50.7 (7.5 − 341.9)
Older (aged ≥ 50 years)		(*n* = 238)		(*n* = 248)			
Low-risk group	−	49	20.6	52	21.0	1.0	1.0
Medium-risk group	−	34	14.3	94	37.9	0.4 (0.2 − 0.7)	0.4 (0.2 − 0.8)
High-risk group	−	87	36.6	70	28.2	1.3 (0.8 − 2.2)	1.2 (0.6 − 2.3)
Low-risk group	+	20	8.4	8	3.2	2.5 (1.0 − 6.2)	3.9 (1.4 − 10.6)
Medium-risk group	+	14	5.9	18	7.3	0.9 (0.4 − 1.9)	1.6 (0.6 − 3.9)
High-risk group	+	34	14.3	6	2.4	5.7 (2.2 − 15.0)	10.2 (3.6 − 28.4)

^1^Low-risk group: individuals carrying 0–1 risk genotypes; medium-risk group: individuals carrying 2 risk genotypes; and high-risk group: individuals carrying 3 or 4 risk genotypes.

^2^ Adjusted for age, sex, and smoking and drinking status.

**Table 6:** **Joint effect of HPV16 serology and combined risk genotypes of *TNF***-***α *SNPs on risk of OSCC**, **stratified by smoking**

**Risk groups**^**1**^	**HPV16 status**	**Patients**		**Controls**		**Adj. OR (95% CI)**^**2**^	
		***(n = 325)***		***(n = 335)***			
		**No.**	**%**	**No.**	**%**	**OSCC**	**SCCOP**
Never-smokers		(*n* = 99)		(*n* = 96)			
Low-risk group	−	15	15.2	32	33.3	1.0	1.0
Medium-risk group	−	17	17.2	38	39.6	1.0 (0.4 − 2.3)	1.0 (0.2 − 3.1)
High-risk group	−	21	21.2	18	18.8	2.3 (1.0 − 5.9)	2.5 (1.0 − 8.6)
Low-risk group	+	10	10.1	5	5.2	5.4 (1.5 − 19.4)	11.0 (2.6 − 46.8)
Medium-risk group	+	11	11.1	1	1.0	28.4 (3.2 − 250.2)	51.6 (5.4 − 496.8)
High-risk group	+	25	25.3	1	2.1	35.6 (7.0 − 180.3)	67.4 (12.0 − 378.5)
Ever-smokers		(*n* = 226)		(*n* = 239)			
Low-risk group	−	46	20.4	49	20.5	1.0	1.0
Medium-risk group	−	36	15.9	91	38.1	0.4 (0.2 − 0.7)	0.5 (0.2 − 1.0)
High-risk group	−	90	39.8	65	27.2	1.5 (0.9 − 2.6)	1.5 (0.8 − 2.9)
Low-risk group	+	17	7.5	8	3.4	2.2 (0.8 − 5.6)	3.6 (1.3 − 10.0)
Medium-risk group	+	12	5.3	20	8.4	0.7 (0.3 − 1.5)	1.2 (0.5 − 3.1)
High-risk group	+	25	11.1	6	2.5	4.8 (1.8 − 12.9)	7.4 (2.6 − 21.4)

^1^Low-risk group: individuals carrying 0–1 risk genotypes; medium-risk group: individuals carrying 2 risk genotypes; and high-risk group: individuals carrying 3 or 4 risk genotypes.

^2^Adjusted for age, sex, and smoking and drinking status.

### Association of combined risk genotypes of four TNF-α SNPs with tumor HPV16-positive SCCOP

To further confirm the modifying effects of *TNF*-α promoter variant on risk of HPV16-associated SCCOP, we assessed the association of the combined risk genotypes with HPV16-positive SCCOP patients based on tumor HPV16 status instead of HPV16 serology. Of 188 SCCOP patients, we determined the tumor HPV16 status among 176 SCCOP patients whose specimens were available for such a testing. We found that the combined genotypes of four variants were significantly associated with tumor HPV16-positive SCCOP patients, and the patients in high-risk group were 5.4 times more likely to have tumor HPV16-positive SCCOP compared with those in low-risk group (OR, 5.4; 95% CI, 2.5-11.7) (Table 7).

**Table 7:** **Association of the combined risk genotypes of *TNF***-***α *SNPs with tumor HPV16**-**positive SCCOP patients**

**Risk groups**^**1**^	**HPV-positive cases**		**HPV-negative cases**		**Adjusted OR (95% CI)**^**2**^
	***(n = 107)***		***(n = 69)***		
	**No.**	**%**	**No.**	**%**	
Low-risk group	18	16.8	32	46.4	1.0
Medium-risk group	25	23.4	16	23.2	2.7 (1.1-6.4)
High-risk group	64	59.8	21	30.4	5.4 (2.5-11.7)

^1^Low-risk group: individuals carrying 0–1 risk genotypes; medium-risk group: individuals carrying 2 risk genotypes; and high-risk group: individuals carrying 3 or 4 risk genotypes.

^2^Adjusted for age, sex, and smoking and alcohol drinking status.

## Discussion

The results of this study show that *TNF*-*α* variants may individually or, more likely, jointly modulate the risk of HPV16-associated OSCC, particularly for SCCOP, and that the joint effect of combined risk genotypes and HPV16 seropositivity was more pronounced in younger subjects and never-smokers. Moreover, the patients with the combined risk genotypes were more likely to have tumor HPV-positive SCCOP. These results suggest that *TNF*-*α* SNPs may serve as a susceptibility biomarker for HPV16-associated OSCC, especially SCCOP.

The four *TNF*-*α* promoter SNPs were selected for this study because of their putatively functional potential in affecting TNF-α production [15,16]; of which *TNF*-*α* -308G > A has been studied mostly in association with OSCC risk. Consistent with our results, several previous studies found a significant increase in risk associated with carrying the minor A allele [22,24,25], while two other studies reported a significantly decreased risk associated with the minor allele [20,26]. Along with the small number of subjects included, differences in ethnicity with different environmental backgrounds may account for the inconsistencies in the findings from these studies. For example, the two studies that reported a decreased risk of OSCC were conducted in the East Asian region, where chewing betel quids, smoking, and drinking are still the predominant risk factors for OSCC [20,26].

None of these published studies has examined the association in the context of HPV infection. For HPV-associated cancer, several studies have reported that the minor A allele of SNP-308 represents an increased risk of HPV infection and HPV-associated cervical cancer from various ethnic groups [17,29]. A meta-analysis based on 2298 cases of cervical cancer and 1903 controls from eight study populations showed a summary OR of 1.31 (95% CI, 1.14–1.52); that analysis compared individuals carrying the GA/AA genotypes with those carrying the GG genotype of *TNF*-*α*-308, in which further stratification analysis by ethnicity showed that the risk remained significant among both Caucasians and Asians [30].

In the present study, we showed that the risk of HPV16-associated OSCC was modified by each of the selected *TNF*-*α* SNPs and that a greater OR for the combined risk genotypes suggests a joint effect of *TNF*-*α* SNPs and HPV16 seropositivity on risk of OSCC, particularly SCCOP. More specifically, head and neck cancer risk associated with smoking, alcohol drinking, and HPV16 infection has been shown to differ by tumor sites, with HPV16 infection being the strongest risk factor for SCCOP and smoking and drinking being the strongest risk factors for oral cavity cancers [31]. These findings are also in accordance with our findings in the present study that the joint effect of HPV16 infection and *TNF*-*α* SNPs was particularly pronounced for SCCOP as opposed to oral cavity cancers. Moreover, such a joint effect was more evident in never-smokers. These results suggest that smoking may not play a major role in HPV-associated OSCC, which may instead be modulated by genetic factors such as *TNF*-*α* SNPs. This hypothesis is further supported by the evidence that HPV16 is an independent risk factor for SCCOP regardless of smoking or drinking status [31]. In addition, we found a greater modifying effect of *TNF*-*α* SNPs on HPV-associated OSCC in young subjects than in older subjects, suggesting an early-age onset of HPV-associated OSCC, a theory that not only supports inherited susceptibility to OSCC but also is consistent with the increasing prevalence of oral HPV in young populations. In general, these results may indicate a potential interaction between *TNF*-*α* SNPs and HPV16 infection that increases the risk of OSCC, although this hypothesis needs to be evaluated in future studies with larger sample sizes. In the current study, although we matched the cases and controls by smoking status, it was necessary to further adjust for these risk factors for their residual effect to reduce the bias in the estimates of the association between *TNF*-*α* SNPs and HPV16-associated OSCC.

The combination of HPV16 infection and carrying three or four risk genotypes of *TNF*-*α* was found in 15.4% of OSCC patients but only in 2.4% of controls. We estimated that individuals who have both factors had 8.5-times greater risk of OSCC compared with HPV16-seronegative individuals categorized as low-risk. Such a cumulative effect is even more evident for SCCOP (OR, 15.8), especially in never-smokers (OR, 67.4). Furthermore, due to the discordance between HPV serological and tumor status, we determined the tumor HPV16 status for SCCOP patients to further explore the association between these *TNF*-*α* promoter variants and susceptibility to HPV-associated SCCOP. We found that the combined risk genotypes were also significantly associated with tumor HPV16-positive SCCOP, indicating that the combined risk genotypes of *TNF*-*α* SNPs may contribute to HPV16-assoctaed SCCOP. It may be possible to use both factors in combination to assess risk for individuals, especially those who never smoked, but larger studies are needed to validate our results.

The current study had some limitations. First, there is possible selection bias from this current case–control study owing to the nature of its hospital-based design. Second, since we only included non-Hispanic whites in the study, our results cannot be generalized to other ethnic groups. Since there were only limited numbers in some subgroups in our stratification analysis, the results need to be confirmed in future studies with larger sample sizes. In addition, it is possible that HPV tumor status among study subjects may have been misclassified in some instances owing to the use of HPV serology. However, in the present study, we took advantage of using HPV serology data for a possible case–control study design to include HPV information for risk evaluation, and further confirmed the association in a subgroup of SCCOP patients with tumor specimens available. Nevertheless, since HPV serological status might not fully reflect actual HPV tumor status, future studies will be needed to establish the correlation of HPV status between sera and tumor tissues. Finally, as we observed, some of the confidence intervals were very wide, indicating lack of precision and a reduced study power owing to the small numbers of the subgroups. This can be improved by a future patient cohort with a larger sample size and tumor HPV information.

We conclude that the variant genotypes of *TNF*-*α* SNPs -308G > A, -857C > T, -863C > A, and -1031T > C may be individually or, more likely jointly, associated with risk of HPV16-associated OSCC, particularly SCCOP, in a non-Hispanic white population. Furthermore, we found that the joint effects were greater among younger individuals and never-smokers. To our knowledge, this is the first study investigating the association of *TNF*-*α* SNPs in promoter region with risk of HPV16-associated OSCC. Future studies with larger sample sizes and more accurate HPV tumor status information are needed to validate these findings.

## Abbreviations

TNF-α: Tumor necrosis factor-alpha; CI: Confidence interval; OR: Odds ratio; PCR-RFLP: Polymerase chain reaction-restriction fragment length polymorphisms; SNP: Single nucleotide polymorphism; OSCC: Oral squamous cell carcinoma; SCCOP: Squamous cell carcinoma of the oropharynx; HPV: Human papillomavirus.

## Competing interests

The authors declare that they have no competing interest.

## Authors’ contributions

LJ participated in the design of the study, data analysis, and manuscript writing. EMS participated in study design, statistical analysis and manuscript writing. YZ and ZH participated in study design, data analysis, and manuscript preparation. XS participated in study design and helped to draft the manuscript. CL participated in statistical analysis and helped to draft the manuscript. QW participated in study design, data analysis, and manuscript writing. GL participated in study design, data analysis and manuscript writing. All authors read and approved the final manuscript.
